# Treating depression with a smartphone-delivered self-help cognitive behavioral therapy for insomnia: study protocol for a parallel group randomized controlled trial

**DOI:** 10.1186/s13063-020-04778-1

**Published:** 2020-10-09

**Authors:** Victoria Ka-Ying Hui, Christy Yim-Fan Wong, Eric Ka-Yiu Ma, Fiona Yan-Yee Ho, Christian S. Chan

**Affiliations:** 1 Department of Psychology, The University of Hong Kong, Pokfulam Road, Hong Kong SAR, Hong Kong; 2 Department of Psychology, The Chinese University of Hong Kong, Shatin, Hong Kong

**Keywords:** Major depression, Insomnia, Cognitive behavioral therapy for insomnia, Sleep disturbance, Smartphone, Internet intervention

## Abstract

**Background:**

Depression is a major public health concern. Emerging research has shown that cognitive behavioral therapy for insomnia (CBT-I) is effective in treating individuals with comorbid insomnia and depression. Traditional face-to-face CBT-I encounters many obstacles related to feasibility, accessibility, and help-seeking stigma. CBT-I delivered via smartphone application could be a potential solution. This paper reports a protocol designed to evaluate the efficacy of a self-help smartphone-based CBT-I, using a waitlist group as control, for people with major depression and insomnia.

**Methods:**

A two-arm parallel randomized controlled trial is conducted in a target sample of 285 non-suicidal Hong Kong Chinese older than 17 years of age with major depression and insomnia. Participants complete an online rapid screening, followed by a telephone diagnostic interview. Those who meet the eligibility criteria are randomized in a ratio of 1:1 to receive either CBT-I immediately or to a waitlist control condition. The CBT-I consists of six weekly modules and is delivered through a smartphone application proACT-S. This smartphone app has been pilot tested and revamped to improve user experience. An online randomized algorithm is used to perform randomization to ensure allocation concealment. The primary outcomes are changes over the measurement points in sleep quality, insomnia severity, and depression severity. The secondary outcomes include changes over the measurement points in anxiety, subjective health, treatment expectancy, and acceptability of treatment. Assessments are administered at baseline, post-intervention, and 6-week follow-up. The recruitment is completed. Important adverse events, if any, are documented. Multilevel linear mixed model based on intention-to-treat principle will be conducted to examine the efficacy of the CBT-I intervention.

**Discussion:**

It is expected that proACT-S is an efficacious brief sleep-focused self-help treatment for people with major depression and insomnia. If proven efficacious, due to its self-help nature, proACT-S may be applicable as a community-based early intervention, thereby reducing the burden of the public healthcare system in Hong Kong.

**Trial registration:**

ClinicalTrials.gov NCT04228146. Retrospectively registered on 14 January 2020.

Major depressive disorder (MDD) poses a significant health problem. In Hong Kong, the 12-month prevalence rate of MDD among adults is estimated at 8.4% [1]. Sleep disturbance, in particular insomnia, is a frequent symptom of MDD. It has been estimated that as many as 90% of patients with depression have sleep quality-related complaints [2]. Furthermore, in addition to being a symptom, insomnia could contribute to MDD symptom maintenance and the development of subsequent major depressive episodes [3]. A review of 21 prospective studies found that insomnia was a predictor of depression and doubled the risk for depression [4]. One longitudinal study of 300 pairs of twins also found that sleep problems predicted depressive symptoms later in life [5]. In Hong Kong, it has been found that sleep disturbances predict suicide attempts among outpatient psychiatric patients [6].

There is strong evidence to support the efficacy and effectiveness of cognitive-behavioral therapy (CBT) in the treatment of both MDD [7] and insomnia [8, 9]. Given their high co-occurrence [10], it has been suggested that treatment for depression should be augmented with a sleep-focused intervention for patients with both symptoms. In fact, sleep treatments, such as cognitive-behavioral therapy for insomnia (CBT-I), have been found to attenuate non-sleep symptoms of MDD [11].

Despite its efficacy in tackling MDD, there exist multiple barriers that prevent patients from receiving CBT in many parts of the world, including Hong Kong. Traditionally, CBT for depression is offered through weekly in-person sessions, delivered by a professional therapist for 10–24 weeks. However, the over-stretched public healthcare system in Hong Kong cannot afford this frequency of care. The median waiting time for new routine cases ranges from 16 to 61 weeks [12], and the typical interval between sessions exceeds 2 to 3 months. Moreover, cultural barriers, especially stigma towards mental illnesses, are also hindering help-seeking behaviors in Hong Kong [13]. Many Chinese patients would see their illness as a source of shame, holding the belief that mental health problem is a result of inferior origins, failure of parents, or the sins of one’s ancestors [14]. Their illness could also cause unfavorable social evaluation and undermine help-seeking behaviors [15].

To overcome these structural and cultural barriers in the treatment of depression, self-help treatments, specifically smartphone-based programs, are proposed to be an economically viable and culturally acceptable mode to deliver empirically supported interventions. In contexts with scarce resources and strong stigma attached to mental illnesses, self-help treatments are feasible alternatives to professionally delivered treatments. With the increasing development and accessibility of technology, Internet and smartphone-based self-help treatments are becoming more readily available and increasingly popular. Emerging evidence suggests that patients with mild to moderate MDD are receptive and respond well to computerized CBT [16]. In a randomized controlled trial (RCT) examining Internet-based CBT for depression, 80% of participants reported satisfaction towards the treatment received, and 74% of participants felt the program was equal to or better than a “real” therapist [17]. Similarly, Internet-based self-help CBT-I was found to significantly reduce both insomnia and depression severity [18]. The effects of internet-based CBT-I on improving insomnia severity, sleep quality, and depression have been supported by multiple meta-analyses (e.g., [19–21]).

Not only are self-help treatments efficacious and effective, they are often preferred over traditional psychotherapy in places where mental health stigma remains prevalent. In a study conducted in Hong Kong examining the 12-month prevalence of major depressive episode [1], large proportions of patients considered self-help as their preferred treatment for depression, over general medical, mental health specialty, and traditional Chinese medicine. CBT-I, in particular, is more acceptable to this population as the treatment for sleep might be perceived as more “physiological” and less “psychological” than treatments for depression [22]. This is confirmed through the high receptiveness of Internet-based self-help CBT-I in a Hong Kong RCT [23]. With the high degree of smartphone penetration (85.8%) in Hong Kong [24], smartphone application might be another promising platform to provide self-help treatment there.

The unprecedented global pandemic of COVID-19 has highlighted the pressing need for remote delivery models of mental health services in the interest of public safety. There is emerging evidence to show that the pandemic and its aftermath have had detrimental effects on mental health [25], especially among people with pre-existing mental disorders and essential workers [26]. In addition, public healthcare systems worldwide face extreme pressure during the pandemic. Many non-urgent outpatient appointments are postponed, and psychiatric admissions are disrupted in response to COVID-19. The increasing need for remote assessments and therapist-client interactions may also accelerate the developments in eHealth/mHealth. Self-help app-based CBT could be utilized to fill the gaps in face-to-face care by increasing public’s access to much needed evidence-based mental health interventions. Therefore, community research that examines the feasibility and potential efficacy of self-help app-based CBT becomes even more relevant in the midst of the present pandemic and future public health crises. The present RCT offers one of the first evaluations of the use of a self-help smartphone app to deliver CBT-I in supporting people with MDD and insomnia in the community during the COVID-19 pandemic.

## Objectives

This paper describes the protocol for a two-arm parallel RCT study that compares the efficacy of a smartphone-based self-help CBT-I with a waitlist control group in treating people with MDD and insomnia in Hong Kong. It is hypothesized that, after the intervention, participants in the CBT-I condition will report a greater decrease in poor sleep quality, depression severity, and insomnia severity than those in the waitlist control condition. It is also hypothesized that the reduction in poor sleep quality, depression severity, and insomnia severity observed in the CBT-I condition will be maintained at 6-week follow-up. Furthermore, it is hypothesized that participants in the waitlist control condition will report a significant decrease in poor sleep quality, depression severity, and insomnia severity after receiving CBT-I.

## Methods

### Trial design

This is a two-arm, with equal randomization (1:1 allocation ratio) parallel, superiority RCT. It compares a self-help smartphone-based CBT-I intervention to a waitlist control condition. The self-help treatment is delivered through “proACT-S,” a smartphone application developed by the corresponding author’s research team. The results from a pilot study showed that proACT-S was efficacious for treating insomnia and was positively received by the users [23]. The current RCT is designed to test the efficacy of proACT-S in treating people with MDD and insomnia. Participants’ change in depressive and insomnia symptoms after the CBT-I intervention will be compared with that of the waitlist control group. The rationale for choosing waitlist control as the comparison condition is twofold: (1) it allows researchers to determine the effect of the intervention against not receiving treatment during the trial period and (2) it ensures that all participants will eventually receive the treatment.

**Fig. 1:**
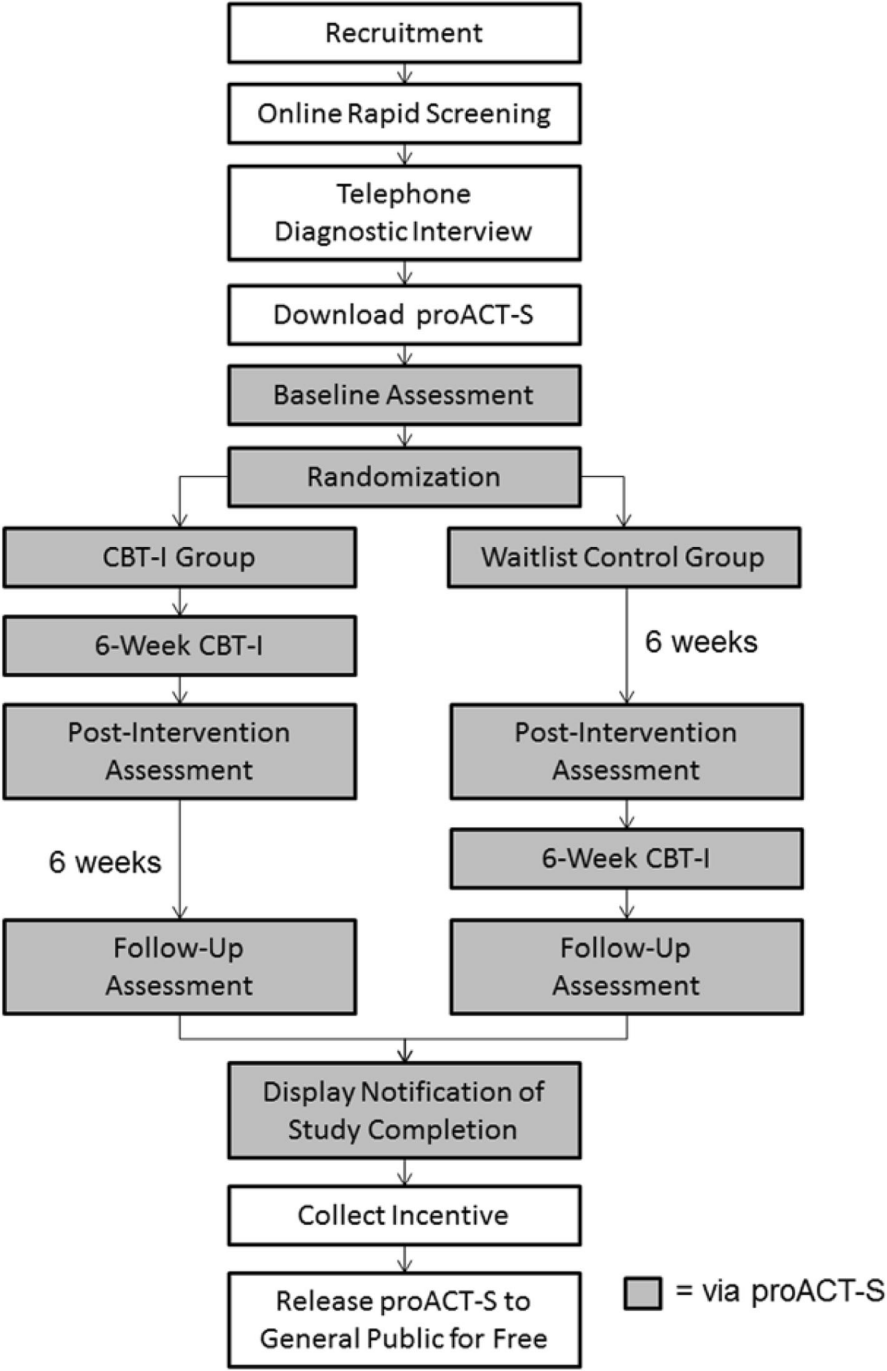
The current RCT study design

Prior to trial entry, participants complete a two-stage screening. Their eligibility is assessed by an online survey, followed by a telephone diagnostic interview. After the two-stage screening, the assessments, randomization, and intervention are carried out via proACT-S. The research design is summarized in Fig. 1. The full SPIRIT checklist is provided as Additional file 1.

### Study setting

This RCT is conducted in Hong Kong, where the 12-month prevalence of MDD among adults was estimated at 8.4% [1], and the weighted prevalence of insomnia among adults was 39.4% [25].

### Eligibility criteria

#### Inclusion criteria

To participate in this study, participants must fulfill the following inclusion criteria: (1) Hong Kong residents; (2) age ≥ 18 years; (3) sleep disturbance causes distress or impairment in social, occupational, and other important areas of functioning for at least three nights per week for at least 3 months; (4) Insomnia Severity Index [27] score ≥ 8; (5) Patient Health Questionnaire (PHQ-9 [28];) score ≥ 10; (6) being able to read Chinese and type Chinese or English; (7) have a smartphone device (iOS or Android operating system) with Internet access; (8) have a regular email address; (9) willing to give informed consent and comply with the trial protocol; (10) difficulty initiating sleep, maintaining sleep, or early morning awakening with inability to return to sleep at least once in the past 2 weeks; and (11) fulfilling International Statistical Classification of Diseases and Related Health Problems—Tenth Revision (ICD-10) diagnosis of depression (F32.00, F32.01, F32.10, F32.11, F32.2).

#### Exclusion criteria

Participants are excluded if they meet the following exclusion criteria: (1) Beck Depression Inventory-II (BDI-II) suicidal ideation score ≥ 2; or (2) receiving psychological treatment at least once per month; or (3) former proACT-S pilot clinical trial participants; or (4) currently taking prescribed psychiatric drugs such as antidepressants, tranquilizers, and sleeping pills regularly; or (5) carrying a diagnosis of psychosis or schizophrenia; or (6) participating in any other academic studies or clinical trials related to insomnia and/or depression; or (7) having current suicidal plans or acts or have had suicidal plans or acts within the past 12 months.

#### Withdrawal criteria

Participants are withdrawn if, during the main study trial, they (1) have concurrent psychological treatment at least once per month; or (2) are taking prescribed psychiatric drugs such as antidepressants, tranquilizers, sleeping pills regularly; or (3) are being diagnosed with psychosis or schizophrenia; or (4) are participating in any other academic studies or clinical trials related to insomnia and/or depression; or (5) have suicidal ideations defined as scoring ≥ 2 on the BDI-II suicidal ideation item; or (6) have experienced serious diseases, significant life events, hospitalization, or fatalities; or (7) withdraw their consent; or (8) do not complete each assessment within 2 weeks, do not submit consent within 2 weeks after proACT-S personal account registration, or do not log into proACT-S within 2 weeks after random group assignment. In addition, participants in the waitlist control group who fail the cross-condition contamination check are withdrawn. Participants in the waitlist control group are withdrawn if (1) they have viewed part or all treatment module content as shown by people who are undergoing this study’s treatment or who have completed this study’s treatment, or who are very familiar with proACT-S (except the proACT-S project team), or (2) they have watched part or all of the treatment module videos included in proACT-S, or (3) they have completed part or all of the treatment module homework in proACT-S.

### Screening measures

A two-stage screening is set up to ascertain whether or not participants fulfill the eligibility criteria. Stage 1 is an online rapid screening and is conducted in the Chinese language. Participants are screened for the aforementioned inclusion criteria 1 to 9, as well as for the aforementioned exclusion criteria 1 to 6. Demographic information is collected, including Hong Kong residency, age, predominant sleep complaints, and the extent of the resulting distress or impairment in social, occupational, and other important areas of functioning. Participants are asked if they are able to read Chinese and type Chinese or English, have a smartphone device (iOS or Android operating system) with Internet access, and have a regular email address. Participants’ severity of insomnia symptoms is measured by the Insomnia Severity Index [29], and each item is rated on a 4-point Likert scale, ranging from “not at all” to “extremely.” It has been validated in Hong Kong samples [30, 31] and has high internal consistency (Cronbach’s alpha = .81 [31]). Participants’ severity of depressive symptoms is measured by the PHQ-9, and each item is rated on a 4-point Likert scale, ranging from “not at all” to “nearly every day.” It has been validated in Hong Kong samples [32] and has high internal consistency (Cronbach’s alpha = .82 [32]). Participants’ suicidal ideation is assessed by BDI-II item 9. Participants are asked to indicate whether they are receiving any concurrent psychological treatment at least once per month, whether they had participated in the proACT-S pilot clinical trial, whether they are currently taking prescribed psychiatric medication regularly, whether they are diagnosed with psychosis or schizophrenia, and whether they are participating in any other academic studies or clinical trials related to insomnia and/or depression.

Stage 2 is a telephone diagnostic interview screening and is conducted in Cantonese. It is used to assess clinical depression and insomnia, which correspond to inclusion criteria 10 and 11. The clinical insomnia inclusion criterion is developed with reference to the Diagnostic and Statistical Manual of Mental Disorders—Fifth Edition (DSM-5 [33]) insomnia disorder diagnostic features. Difficulty initiating sleep is defined by a subjective sleep latency greater than 30 min, difficulty maintaining sleep is defined by frequent awakenings or problems returning to sleep within 30 min after awakenings, and early morning awakening is defined by awakening at least 30 min before the scheduled time and the total sleep time before the awakening is less than 6.5 h. The clinical depression inclusion criterion is based on the modified Chinese version of the Revised Clinical Interview Schedule (CIS-R [34]) algorithm for ICD-10 Mild depressive episode without somatic symptoms (F32.00). The timeframe for items measuring ICD-10 depressive episode has changed from “in the past week” to “in the past 2 weeks” to tap the 2-week requirement for DSM-5 depressive episode diagnostic criteria. In order to meet the criteria of mild depressive episode without somatic symptoms, the participants need to fulfill the following four conditions: (1) having a minimum of 2-week duration of depressive episode; (2) endorsing at least two prominent symptoms of depression—depressed mood, loss of interest, fatigue—in the past 2 weeks; (3) endorsing at least two of the other common depressive symptoms—reduced concentration, reduced self-esteem, ideas of guilt or worthlessness, pessimism about future, suicidal ideation, disturbed sleep, change in appetite with corresponding weight change—in the past 2 weeks; and (4) obtaining a norm-based mental component score of ≤ 45 in the 12-item Short-Form Health Survey Version 1 (SF-12 Version 1) [35]. Because no epidemiological research has been conducted to assess the screening utility of the SF-12 Version 1 mental health component scale for diagnosable depression in Hong Kong Chinese population, the cutoff score of ≤ 45 was chosen with reference to a study that assessed its diagnostic accuracy to predict depression in the general Australian population [36]. In addition, stage 2 telephone diagnostic interview screening is used to reject participants who meet the aforementioned exclusion criteria 2 to 7.

### CBT-I intervention

The self-help CBT-I treatment content is based on the Chinese translated version of a well-established CBT-I treatment manual entitled “Insomnia: A Clinician’s Guide to Assessment and Treatment” [37]. CBT-I aims at changing dysfunctional cognitive beliefs and maladaptive behaviors that contribute to the maintenance of insomnia. The self-help CBT-I treatment is delivered in the Chinese language in six consecutive weekly modules via proACT-S. The duration of each module is around 45 to 60 min. The content of each treatment module is displayed in Table 1.

**Table 1:** Content of each weekly CBT-I treatment module

Week 1	Content	Treatment overview
		Predisposing, precipitating, and perpetuating factors of insomnia
	Homework	Dysfunctional beliefs and attitudes about sleep assessment
Week 2	Content	Sleep hygiene
	Homework	Sleep hygiene assessment
Week 3	Content	Basic facts about sleep
		Relaxation therapy
	Homework	Diaphragmatic breathing relaxation (daily practice)
		Sleep diary (at least three diaries per week)
Week 4	Content	Sleep restriction
		Stimulus control
	Homework	Sleep diary (at least three diaries per week)
		Sleep restriction (sleep efficiency ≥ 90%; 15 min per week maximum)
Week 5	Content	Cognitive restructuring
		Constructive worry
		Cognitive distortions
	Homework	Thought record
Week 6	Content	Integration and review of all treatment content
		Shift work

proACT-S has been pilot tested in a sample of 32 Hong Kong Chinese participants and, later, revamped to improve user experience. One current recommendation for engaging app users was to reduce the amount of text and, instead, to use videos to deliver treatment content [38]. Therefore, animations were added to the text-based materials to enhance user experience. Following the literature recommendations [39, 40], more interactive components were incorporated into the app. In particular, proACT-S now provides (a) a clear timeline to indicate the start date for each assessment and treatment module, (b) indicators for module and assessment completion, (c) more prompts for users to follow in completing the weekly homework, (d) a modifiable sleep diary that accommodates the sleep pattern of shift-workers, and (e) individualized homework feedback.

Reasons for discontinuation of CBT-I may include, but are not limited to, the following: (1) participants’ decision to discontinue treatment at any time for any reason and (2) principal investigator’s decision to terminate treatment for the participants’ safety reasons at any time.

### Outcomes

#### Primary outcomes

##### Depression severity

The 20-item Center for Epidemiologic Studies Depression Scale [41] is used to measure participants’ severity of depressive symptoms during the past week. Each item is rated on a 4-point Likert scale, ranging from “less than 1 day” to “5–7 days.” It has been validated in a Hong Kong sample and has high internal consistency (Cronbach’s alpha = .85 [42]).

##### Insomnia severity

The 7-item Insomnia Severity Index [29] is used to assess participants’ severity of insomnia symptoms and the associated daytime impairment over the past 2 weeks. Each item is rated on a 4-point Likert scale, ranging from “not at all” to “extremely.” It has been validated in Hong Kong samples [30, 31] and has high internal consistency (Cronbach’s alpha = .81 [31]).

##### Sleep quality

The 19-item Pittsburgh Sleep Quality Index [43] is used to measure participants’ sleep quality and disturbances during the past month. It has seven components, namely, sleep quality, sleep latency, sleep duration, habitual sleep efficiency, sleep disturbance, use of hypnotics, and daytime dysfunction. Each subscale is converted to a scale of 0 to 3. It has been validated in Hong Kong samples [25] and has good reliability (Cronbach’s alphas range from .59 to .63 [44]).

#### Secondary outcomes

##### Subjective health

The SF-12 Version 1 is used to measure participants’ subjective physical and mental health status. The SF-12 Version 1 is scored using the recommended norm-based scoring with a mean of 50 and a standard deviation of 10 in the general US population [35]. It has been validated in Hong Kong samples [25, 45].

##### Anxiety

The 7-item Hospital Anxiety and Depression Scale—Anxiety subscale [46] is used to measure participants’ severity of anxiety symptoms during the past week. Each item is rated on a 4-point Likert scale. It has been validated in Hong Kong samples [25, 47] and has high reliability (Cronbach’s alpha = .8 [47]).

##### Treatment expectancy

The 6-item Credibility/Expectancy Questionnaire [48] has been modified to measure participants’ cognitively and affectively based expectancy towards the treatment. The original phrase “trauma symptoms” has been changed to “depressive symptoms” or “insomnia symptoms.” Four items are rated on a 9-point Likert scale, ranging from “not at all” to “very.” The remaining two items are rated on a 11-point Likert scale, ranging from “0%” to “100%.” It has been validated in cognitive behavioral therapeutic interventions for people with depression [49] or insomnia [50], and it has high internal consistency (Cronbach’s alphas range from .81 to .96).

##### Acceptability of treatment

The 26-item modified Participant Acceptability/Usability Rating Scale [51] is used to measure participants’ evaluation of the treatment via proACT-S on a 3-point Likert scale, ranging from “agree” to “disagree.” It has been validated in a pilot CBT-I study in Hong Kong [52].

##### Demographics

Information about participants’ age, education level, marital status, occupation, and gender is obtained from the stage 1 online rapid screening.

##### Clinical comorbidity

The number of participants with a current diagnosis of four major comorbidities (generalized anxiety disorder, phobias, obsessive-compulsive disorder, and panic disorder) are estimated from the modified Chinese version of the CIS-R [34] administered in the stage 2 telephone diagnostic interview screening. The modified Chinese version of the CIS-R [34] measures the ICD-10 diagnoses related to generalized anxiety disorder (F41.1), phobias (F40.00, F40.01, F40.1, F40.2), obsessive-compulsive disorder (F42), and panic disorder (F41.0).

Primary outcome assessments take place at baseline, post-intervention, and 6-week follow-up for both CBT-I and waitlist control conditions. The schedule of enrolment, interventions, and assessments is summarized in Table 2.

**Table 2:** The Schedule of Enrolment, Interventions, and Assessments

	Screening	Baseline		Allocation	Post intervention		6-week follow-up	
Timepoint (week)	− 12	0		1	7		13	
**Enrolment:**								
Eligibility screening	X							
Informed consent	X	X						
Allocation				X				
**Interventions:**								
CBT-I		X		X	X		X	
Waitlist control (WLC)		X		X	X		X	
**Assessments:**								
*Primary outcome measures*		CBT-I	WLC		CBT-I	WLC	CBT-I	WLC
Depression severity (CES-D)		X	X		X	X	X	X
Insomnia severity (ISI)		X	X		X	X	X	X
Sleep quality (PSQI)		X	X		X	X	X	X
*Secondary outcome measures*		CBT-I	WLC		CBT-I	WLC	CBT-I	WLC
Subjective health (SF-12 Version 1)		X	X		X	X	X	X
Anxiety (HADS-A)		X	X		X	X	X	X
Treatment expectancy (modified CEQ)		X	X		X			X
Treatment acceptability (modified PARS)		X	X		X			X
Demographics	X							
Clinical comorbidity	X							
*Post-screening eligibility assessments*		CBT-I	WLC		CBT-I	WLC	CBT-I	WLC
Withdrawal criteria					X	X	X	X
Cross-condition contamination check						X		X

*CES-D* Center for Epidemiologic Studies Depression Scale, *ISI* Insomnia Severity Index, *PSQI* Pittsburgh Sleep Quality Index, *HADS-A* Hospital Anxiety and Depression Scale—Anxiety subscale, *CEQ* Credibility/Expectancy Questionnaire, *PARS* Participant Acceptability/Usability Rating Scale

### Participant timeline

Individuals interested in participating are invited to complete a two-stage screening. In stage 1 online rapid screening, they complete an online survey to review their eligibility. Those who have passed the online rapid screening will receive a link within the Qualtrics platform to sign up for the stage 2 telephone diagnostic interview screening. These telephone interviews are conducted by project assistants, who have been trained and supervised on the administration of the modified Chinese version of the CIS-R [34].

Individuals who do not meet the stage 2 telephone diagnostic interview screening eligibility criteria will receive a list of references related to insomnia and depression produced by the Department of Health and the Social Welfare Department under the Government of the Hong Kong SAR. Information regarding crisis hotlines and integrated community centers for mental wellness are provided to those currently with suicidal plans or acts, or those who have had suicidal plans or acts within the past 12 months.

Individuals who have passed the two-stage screening are directed to download proACT-S either from App Store or Google Play. Eligible participants are guided to register for their own personal accounts to enter the main study trial. They then complete the baseline assessment via proACT-S. After that, they are randomly assigned in a 1:1 ratio either to CBT-I condition or waitlist control condition.

Participants in the CBT-I condition start the 6-week CBT-I immediately after randomization, complete the post-intervention assessment right after they finish the treatment, and complete the follow-up assessment 6 weeks after the post-intervention assessment. Participants in the waitlist control group wait for 6 weeks without the proACT-S intervention and then complete the post-intervention assessment 6 weeks after the baseline assessment. The waitlist control participants start CBT-I (equivalent to that of the CBT-I group) immediately after completing the post-intervention assessment, and they complete the follow-up assessment right after finishing the 6-week CBT-I.

**Fig. 2:**
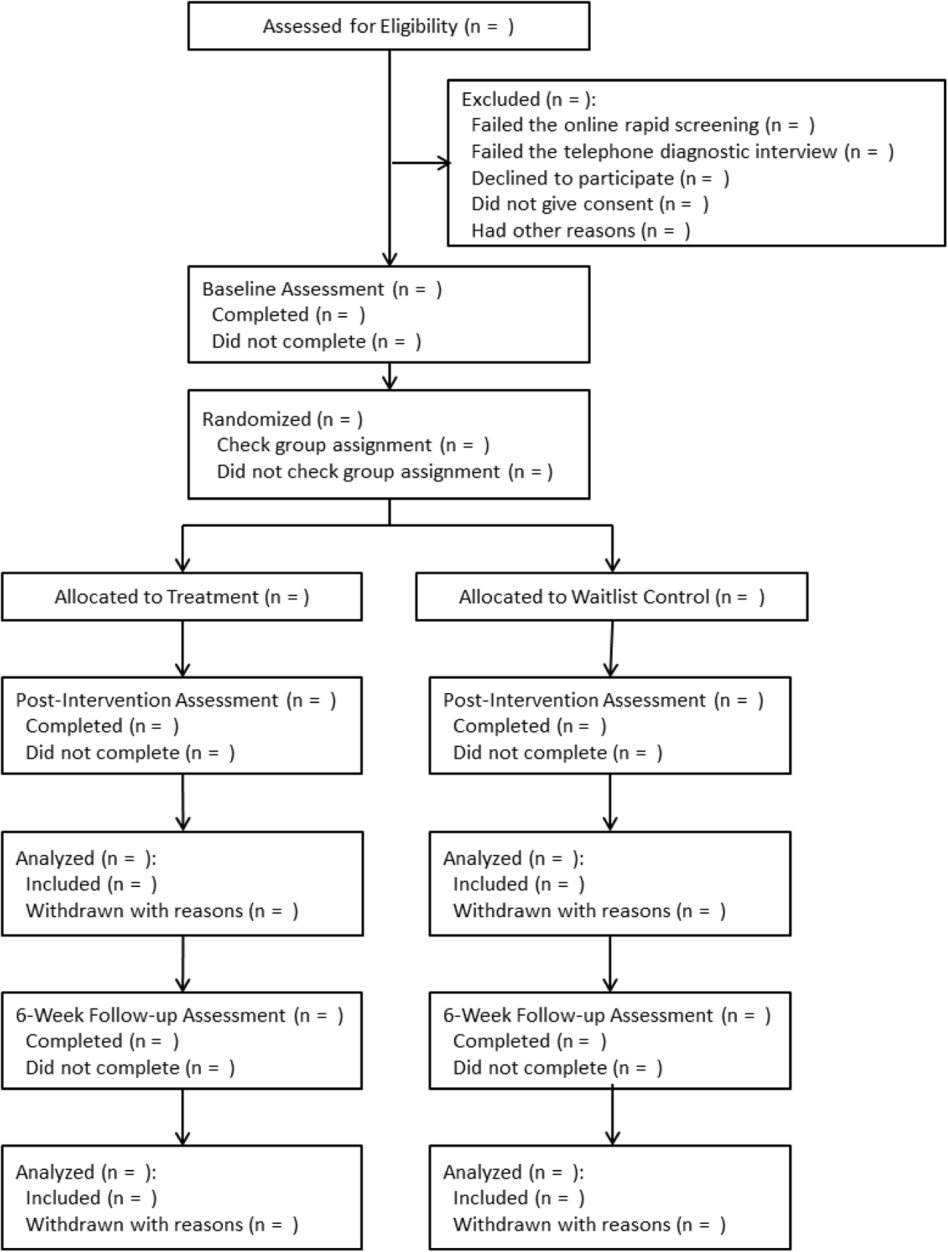
Proposed flow of participants

Emails and WhatsApp reminders are sent to participants to increase their engagement and to enhance treatment compliance. Upon completion of the study, each eligible participant receive cash coupons of HKD $100 as a token of appreciation for their participation. The proposed flow of participants is displayed in Fig. 2.

### Sample size

The most updated meta-analysis of 28 self-help CBT-I RCTs [19] showed a significant treatment effect in alleviating depressive symptoms (Hedges’ *g* = 0.35) and insomnia symptoms (Hedges’ *g* = 0.79). It also found that the mean cumulative study attrition rates were 21.25% (SD = 15.31%) and 18.4% (SD = 18.21%) in the treatment and the waiting list/routine care/no treatment/psychoeducation groups, respectively. Hence, a conservative small effect size (Cohen’s *f* = 0.2) and an attrition rate of 30% are estimated for the present study.

The sample size calculation for each of the three primary outcomes (i.e., insomnia severity, poor sleep quality, and depression severity) is based on mixed ANCOVA using GPower 3.1. To conduct a 2 (condition: CBT-I versus waitlist control) × 2 (assessment: baseline versus post-intervention) mixed ANCOVA with an alpha value set at 0.05, two-sided, 80% power to detect a small effect of 0.2 (Cohen’s *f*) between two groups while controlling for 12 covariates (demographics, clinical comorbidity, treatment expectancy, and acceptability of treatment), a total sample size of 199 will be required. In order to account for 30% attrition, a total sample of 285 participants will be sufficient to detect a small effect size (Cohen’s *f* = 0.2) for the difference in the change in each of the three primary outcomes (i.e., insomnia severity, poor sleep quality, and depression severity) from baseline to post-intervention between CBT-I condition and waitlist control condition with a two-sided 5% significance level and a power of 80%.

### Recruitment

Participants are recruited on a rolling basis through posters, web-based advertisements, departmental website, social media, and institutional mass mailing. A “proACT-S” Facebook page and a “proACT-S” Instagram account have also been set up for this project, where they contain the recruitment poster and local news articles related to insomnia and depression. Recruitment began in March 2019 and was completed in September 2020.

### Allocation

One week following the completion of the baseline assessment, participants are randomly assigned in a ratio of 1:1 either to the CBT-I group or the waitlist control group using an online randomized algorithm (https://www.php.net/manual/en/function.random-int.php). The group assignment follows the simple randomization procedure and is completely automated to ensure allocation concealment.

### Blinding

The principal investigator is blind to both the outcome assessment and group assignment. The research team is blind to the outcome assessment because all three assessments are self-reported and carried out via the smartphone app, but is not blind to the group assignment as research members need to send emails and WhatsApp reminders to the participants to enhance treatment compliance. Efforts are made to minimize participants’ knowledge of treatment allocation by informing participants that the treatment start date is randomly assigned. Statistical analyses will be carried out by a researcher blind to the study protocol. In the unlikely event of a critical incident, the research team, as well as the principal investigator, will be authorized to break the blind and carefully record it on the Case Report Form.

### Involvement of external parties

Psychiatric staff who were involved in validating the Chinese version of the CIS-R in Hong Kong were consulted regarding the telephone diagnostic interview training, item selection, and ICD-10 scoring algorithm. Clinical psychologists specialized in sleep disorders and potential end-users were invited by the research team to provide feedback regarding the study design (e.g., the measures, recruitment strategies, Facebook page), as well as proACT-S revamp and beta testing.

### Possible harms

Adverse events, such as increased suicidal risk and hospital admission, study attrition rates, and significant deterioration in primary outcomes, are monitored and recorded throughout the study trial. Treatment discontinuation decision may be made at the discretion of the principal investigator, for reasons concerning participants’ safety. The proportion of participants experiencing any adverse events in each group will be reported in the results of this RCT study.

### Data analysis plan

The principle of intention-to-treat analysis will be adopted, in which all randomized participants with missing observations, from lost to follow-up or incomplete outcome assessments, will be handled by multiple imputation [53]. Missing data is assumed to be 5%.

Chi-square tests and independent *t* tests will be conducted to examine if participants in the CBT-I and waitlist control conditions differ in terms of demographic characteristics, clinical comorbidity, and baseline outcome scores. Multilevel linear mixed model based on the intention-to-treat principle will be used to calculate between-condition mean differences in the primary outcome changes in sleep quality, insomnia, and depression severity from baseline to post-intervention assessments. Multilevel linear mixed model based on the intention-to-treat principle will also be used to analyze secondary outcomes of the baseline to post-intervention change in subjective health and anxiety between CBT-I and waitlist control conditions. Clustering effect, significant baseline differences in demographics, clinical characteristics, and outcome variables will be adjusted for. Pair-sample *t* tests based on the intention-to-treat principle will be used to analyze changes in primary outcomes, subjective health, and anxiety obtained at post-intervention and 6-week follow-up separately for each condition. All comparisons are planned with a 5% two-sided significance level. Bonferroni adjustment will be applied.

### Ethics and dissemination

Consent is obtained electronically for the stage 1 online rapid screening, verbally for the stage 2 telephone diagnostic interview screening, and via the smartphone app proACT-S for the 6-week CBT-I intervention. The consent form explains that participation is voluntary; individuals could withdraw from the study at any time without any consequences.

Participants directly enter their data via Qualtrics during the stage 1 online rapid screening and via proACT-S during the main trial study. Manual data entry from trained project assistants (i.e., telephone interviewers) is required for recording participants’ stage 2 telephone diagnostic interview screening data. A Qualtrics survey based on the modified Chinese version of the CIS-R [34] was created to enable data entry, coding, and storage, in addition to standardizing the administration of the stage 2 screening across different trained telephone interviewers. Participants’ personal data are sent to the electronic server located at The University of Hong Kong and protected using dual encryption. The principal investigator and the research team are given access to the dataset. Participants’ research files may be reviewed by the Human Research Ethics Committee of the University of Hong Kong in order to check that the study is being carried out correctly. Participants will not be identified by name in any report of the completed study. Their personal data will be kept for five years after the publication of the first original research paper, and research data without personal identifiers will be stored for long term retention at the Community Action Research Laboratory at The University of Hong Kong.

This protocol will be published in an open-access journal for public access. Researchers interested in using the de-identified dataset to test novel hypotheses could contact the corresponding author and submit a data proposal form to be reviewed by the research team. The study findings will be disseminated in peer-reviewed publications and conference presentations. Executive summaries will be made available on the proACT-S project website and disseminated to other organizations such as the funding agency and local news agencies.

## Discussion

This two-arm, parallel randomized controlled clinical study aims to compare the efficacy of a self-help CBT-I smartphone application, proACT-S, with a waitlist control group for people with MDD and insomnia. It is expected that proACT-S would be an efficacious brief sleep-focused self-help treatment for people with MDD and insomnia. If proven efficacious, due to its self-help nature, proACT-S may be applicable as a community-based early intervention, thereby reducing the burden of the public healthcare system in Hong Kong. Additionally, this may encourage wider dissemination and utilization of proACT-S within healthcare settings as an alternative treatment option for depression in the Chinese-speaking regions and communities, who might be less receptive to mental health treatments due to stigmatization.

Although the sample recruitment may be biased towards those who are reluctant to seek help from healthcare professionals, this study will provide valuable evidence regarding the utility of proACT-S as a preventive tool for individuals considering face-to-face therapy or who are receptive to lifestyle medicine for depression and insomnia in Hong Kong. The Hong Kong anti-extradition bill protest began in June 2019 (i.e., 3 months after the recruitment). The social turmoil arising from the protests is likely to trigger a mental health crisis, as revealed by a longitudinal survey finding that nearly one in ten people in Hong Kong are suffering from probable depression [28]. It is believed that many participants in this study will be affected by the social turmoil, which may have a bearing on the CBT-I efficacy. The unstable socio-political situations may make participants more vulnerable to stress-related disorders, such as mood and sleep disorders [26]. Therefore, the social turmoil may undermine the magnitude of reduction in poor sleep quality, depression severity, and insomnia severity after completing this study’s treatment. Nonetheless, it is hoped that utilizing the self-help CBT-I via proACT-S would bring some relief to most participants during the turbulent political times in Hong Kong.

The COVID-19 pandemic would also likely pose an influence on this RCT study. For example, fear of contracting the virus and of spreading it to loved ones may further exacerbate one’s pre-existing vulnerability. Despite these limitations, evaluation of the feasibility, acceptability, and efficacy of self-help CBT-I remains critical for informing future remote mental health service delivery. proACT-S could serve as an accessible and remote resource for participants to practice self-care every day. For example, self-monitoring through a daily sleep diary and thought record could help participants recognize their sleep habits and maladaptive thoughts, as well as the resulting effects on their depression and insomnia. It is hoped that proACT-S would be beneficial for supporting self-care and overall mental health of people with MDD and insomnia during and after the COVID-19 pandemic.

## Trial status

Current protocol is EA1810026, version 4, which was last updated on 15 February 2020. Recruitment began on 19 March 2019 and was completed on 4 September 2020.

## Supplementary information

**Additional file 1.** SPIRIT 2013 Checklist: Recommended items to address in a clinical trial protocol and related documents.

## Data Availability

Not applicable.

## Abbreviations

BDI-II: Beck Depression Inventory II
CBT-I: Cognitive behavioral therapy for insomnia
CIS-R: Revised Clinical Interview Schedule
DSM-5: Diagnostic and Statistical Manual of Mental Disorders—Fifth Edition
ICD-10: International Statistical Classification of Diseases and Related Health Problems—Tenth Revision
MDD: Major depressive disorder
RCT: Randomized controlled trial
SF-12 Version 1: 12-item Short-Form Health Survey Version 1
